# Recent Progress in Stem Cell Modification for Cardiac Regeneration

**DOI:** 10.1155/2018/1909346

**Published:** 2018-01-16

**Authors:** Heiko Lemcke, Natalia Voronina, Gustav Steinhoff, Robert David

**Affiliations:** ^1^Department of Cardiac Surgery, Reference and Translation Center for Cardiac Stem Cell Therapy (RTC), University of Rostock, Schillingallee 69, 18057 Rostock, Germany; ^2^Department Life, Light and Matter of the Interdisciplinary Faculty, University of Rostock, Albert-Einstein Straße 25, 18059 Rostock, Germany

## Abstract

During the past decades, stem cell-based therapy has acquired a promising role in regenerative medicine. The application of novel cell therapeutics for the treatment of cardiovascular diseases could potentially achieve the ambitious aim of effective cardiac regeneration. Despite the highly positive results from preclinical studies, data from phase I/II clinical trials are inconsistent and the improvement of cardiac remodeling and heart performance was found to be quite limited. The major issues which cardiac stem cell therapy is facing include inefficient cell delivery to the site of injury, accompanied by low cell retention and weak effectiveness of remaining stem cells in tissue regeneration. According to preclinical and clinical studies, various stem cells (adult stem cells, embryonic stem cells, and induced pluripotent stem cells) represent the most promising cell types so far. Beside the selection of the appropriate cell type, researchers have developed several strategies to produce “second-generation” stem cell products with improved regenerative capacity. Genetic and nongenetic modifications, chemical and physical preconditioning, and the application of biomaterials were found to significantly enhance the regenerative capacity of transplanted stem cells. In this review, we will give an overview of the recent developments in stem cell engineering with the goal to facilitate stem cell delivery and to promote their cardiac regenerative activity.

## 1. Cardiovascular Disorders in the Modern World

Cardiovascular diseases (CVDs) are the major cause of mortality and disability worldwide. In the United States alone, approximately one million myocardial infarctions (MI) occur yearly, and many of these patients develop heart failure, which is currently diagnosed in five million patients [1–3]. Due to the high number of patients and high-cost treatment, CVDs also represent a serious financial burden [1, 4]. CVDs include various disorders affecting the heart and vessels: coronary heart disease, cerebrovascular disease, peripheral arterial disease, rheumatic heart disease, congenital heart disease, deep vein thrombosis, and pulmonary embolism. Among these, the most frequent cases of tissue ischemia are associated with coronary heart disease, stroke, and peripheral arterial disease, which together account for more than half of all CVDs [4].

Until recently, the heart was suggested to be a terminally differentiated organ incapable of regeneration. However, the most recent findings have proven that at the age of 20 the renewal rate for cardiomyocytes reaches 1%, whereas at 70 it decreases down to 0.4% per year [2, 5]. At the same time, even without diagnosed heart disease, cardiac overload or the aging process are associated with significant loss of cardiomyocytes—up to 20 million yearly (to compare, the left ventricle contains 2–4 billion cardiomyocytes). Furthermore, an acute event such as MI causes loss of billions of cells, reaching 25% of the total heart mass [1]. Since cardiomyocytes are endogenously regenerated in a very limited degree, compensation of this cell loss is achieved by formation of fibrotic scar tissue that does impair heart contractility [2].

## 2. Cell Therapy for Cardiovascular Regeneration—An Alternative Treatment Approach

Currently, there are no efficient pharmaceutical or surgical strategies for the prevention of ischemia-mediated damage and for full regeneration of the injured heart tissue [6]. Besides cardiac resynchronization, angioplasty, or ventricular assist devices, several drugs are applied for the management of hypertension or dyslipidemia and for the control of metabolic symptoms [7]. In particular, all current pharmacological treatments applied in heart failure are principally palliative: they are helpful in improving the quality of life but are not able to change the course of disease. In this regard, the only curative option is heart transplantation. Similarly, in MI treatment, even the most successful developments in surgery are restricted to an improvement of blood supply through manipulation of large vessels [8]. At the same time, one of the key mechanisms for inoperable heart conditions is microangiopathy, where the lack of microcirculation is causing ischemia. Thus, current medical developments are not able to significantly change the course of MI too.

To conclude, the current status of therapy for CVDs is insufficient and development of safe and efficient alternative treatments is necessary. Gene or stem cell therapy and their combination are the major promising strategies thereof. In contrast to currently applied treatments, stem cells have the potential to stimulate and support endogenous mechanisms of cardiac repair and thus provide the basis for full regeneration of damaged heart tissue.

### 2.1. Cell Types Currently Applied

Two main categories of stem cells (SCs) are currently exploited for cardiac regenerative medicine: (1) multipotent adult SCs and (2) pluripotent embryonic SCs (ESCs) and induced pluripotent SCs (iPSCs), where either differentiated derivatives are being explored for transplantation or cells are differentiated in situ after transplantation [9–11]. As potential therapeutics, both these groups carry certain advantages and disadvantages [10, 11].

ESCs and iPSCs share significant benefits: pluripotency, efficient expansion *in vitro*, availability of high cell numbers, and opportunity to create cell banks and off-the-shelf products [12, 13]. In addition, in case of iPSCs, autologous cells for transplantation are available. At the same time, teratoma formation is possible due to either remainders of pluripotent cells in final differentiated cell fraction or impaired in situ differentiation [12]. Moreover, preparation of the final therapeutic product of these cells—either proliferation or differentiation—requires their prolonged culture. This, in turn, can lead to upregulation of miRNAs commonly found in cancers and increase the possibility of genetic and epigenetic abnormalities [11]. Importantly, the use of allogenic ESCs implies possible severe complications due to immune system reaction. In addition, it has been provoking serious ethical and legal debates for decades [11].

Adult SC group consists of different populations of stem and progenitor cells, isolated from various sources, including bone marrow, circulating blood, or solid resident tissues. Most commonly applied cell types are the following: bone marrow-derived mononuclear cells, hematopoietic SCs, endothelial progenitor cells, cardiac SCs (CSCs), and mesenchymal stem cells (MSCs). Their clinical development for the treatment of cardiac patients is very advanced: the most of the clinical translation path is undergone by now. A growing number of preclinical and clinical trials have led to serious positive outcomes within the field of adult stem and progenitor cell transplantation for CVD therapy. First, the therapeutic regeneration using cell products has been demonstrated in several clinical trials (RENEW (NCT01508910), PROCHYMAL (NCT00690066), SCIPIO (NCT00474461), FINCELL (NCT00363324), etc.). In addition, attempts to establish an optimal match between cell product and patient's cohort have been made (PERFECT (NCT00950274). Furthermore, bone marrow-derived progenitor cells were found to positively influence patients with the most extensive MI-induced damage including a low baseline of left ventricular ejection fraction (LVEF) (REPAIR-AMI (NCT00279175), FINCELL, and REGENT (NCT00316381)). For other patients, emerging cell types like CSCs may be suitable which will be defined in planned and ongoing clinical trials [14]. Importantly, the possibility of safe allogenic cell therapy without immunosensitization has been proven for MSCs and CSCs (POSEIDON (NCT01087996), PROCHYMAL (NCT00690066), and ALLSTAR (NCT01458405)), which enables the generation of “off-the-shelf” products.

The main risks related to the adult SC transplantation are rare, usually manageable, and similar for all cell products: immunogenicity and possible occurrence of arrhythmias (the latter particularly for MSCs) [2, 10]. Taken together, the clinical use of adult SCs has proven to be safe for transplantation with a certain evidence of clinical efficacy; thus, further phase II and III trials can be initiated. At the same time, although safety and feasibility of these different cell types have been proven in several clinical trials, the beneficial outcome for cardiac performance is usually very limited [2, 15, 16]. In particular, most successful results have been achieved for CSCs (~10% functional improvement in phase I clinical trial) [17, 18], whereas other commonly applied cell types lead to an average 3–5% beneficial outcome or no positive effect [19].

A new approach for improving cardiac regeneration has been recently described by Luo et al., who generated functionalized microparticles, mimicking stem cell properties [20]. The authors used biodegradable poly(lactic-co-glycolic acid) to encapsulate the secretome of MSCs, followed by coating of these particles with a MSC-derived membrane. Upon transplantation into infarcted mice hearts, these synthetic cell particles demonstrated a regenerative capacity comparable to MSCs. Likewise, the same group utilized cell membranes derived from CSCs to fabricate “synthetic stem cell” products, which were also found to significantly enhance cardiac remodeling and function *in vivo* [21]. The clinical application of these “synthetic stem cell” analogs would overcome the hurdles stem cell therapy is facing, including sufficient storage stability of the cell product, stimulation of an immune reaction, and tumourigenicity caused by cell transplantation [22].

### 2.2. Current Difficulties of Cell Therapy

Analysis of existing trials reveals current problems and challenges, which cell therapy for CVDs is facing. First, despite the fact that most clinical trials have been initiated involving adult SCs, analysis of their results is rather challenging. The main reasons are inconsistency of patients' cohort selection and variability in choice of cell population. Other limitations of currently available adult SC therapeutics are the following: available numbers of fractioned bone marrow-derived cells are low, the replicative capacity of adult SCs in situ is limited compared to ESCs and iPSCs, and adult SCs are restricted to a certain lineage. Moreover, the regenerative capacity of cells declines with age and progenitors mobilized in the body may also lack capability with age [14, 23, 24].

The delivery of sufficient cell numbers to the site of injury also remains a challenging issue. Currently, reported rates of cell retention drop below 5–10% as soon as several minutes to hours post application, regardless of administration routes [25–27]. After delivery, the regenerative potential of cells is often compromised by poor engraftment and survival in the ischemic tissue [28, 29]. These factors may explain, at least in part, why the therapeutic benefit of adult SC application is so limited (~4-5% functional improvement except primary studies of CSCs) [19]. In addition to the poor delivery of sufficient cell numbers, teratogenic and cancerogenic effects remain one of the biggest concerns for ESC and iPSC application (see Section 2.1).

**Figure 1:**
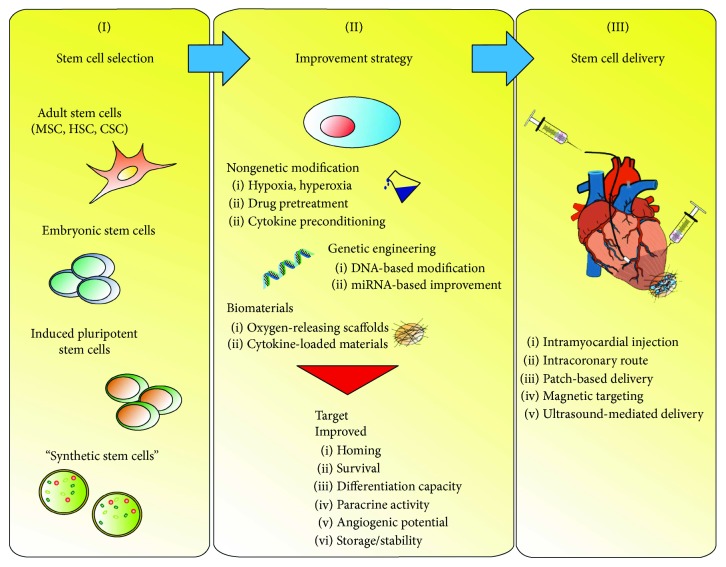
Strategies for improving SC-based therapy in CVD treatment. (I) Multipotent adult SCs and pluripotent stem cells, including embryonic SCs and iPSCs, represent the most widely explored cell types for cardiac regeneration. Novel approaches encompass the generation of synthetic particles (“synthetic stem cells”), mimicking stem cell properties. (II) To enhance their therapeutic activity, multiple strategies have been developed and tested *in vivo*, in some cases reaching clinical trials. While nongenetic modifications are mainly based on the preconditioning with environmental or pharmacological agents, genetic cell engineering utilizes modification on the DNA or posttranscriptional level (miRNA). In addition, the application of cells with supportive biomaterials has proven to greatly increase SC efficiency. The applied strategies positively influence the resistance of SC to the harsh ischemic microenvironment of the damaged heart tissue. Likewise, increased paracrine activity, homing and differentiation capacity, and enhanced proangiogenic activity are common targets for cell improvement. (III) Following successful modification of SC products, optimized administration routes and targeting approaches are developed to ensure proper cell delivery and engraftment.

To conclude, this limited outcome of SC intervention urgently requires improving the therapeutic properties of applied cell types in order to increase their impact on cardiac regeneration (Figure 1).

## 3. Strategies to Improve Cell Therapeutics

### 3.1. Selection of the Optimal Cell Type

Enrichment of transplanted cells for certain cell populations can significantly influence the outcome of therapy. The most illustrative example is selection of cell types from bone marrow based on their properties and surface marker patterns. In particular, uniform isolated cell fractions have demonstrated consistent positive outcomes in several clinical trials (BOOST (NCT00224536), REPAIR-AMI, and FINCELL) versus no functional improvement in many trials involving unselected MNCs (ASTAMI, HEBE, TIME, and Late-TIME). In addition, enrichment of transplanted products for a particular cell type ensures consistency of clinical trials' outcomes and thereby more reliable results. To obtain purified populations of adult SCs from patient's tissue, manual approaches and semiautomatic isolation devices based on magnetic cell sorting have been applied [30].

For resident CSCs, two main categories are currently defined: (1) cardiac progenitors and (2) cardiospheres and cardiosphere-derived cells. To date, eight different CSC subtypes have been identified according to their expression of transcription factors and surface markers, including c-kit and Sca-1 [31].

CSCs expressing the SC factor receptor c-kit are mainly present in the atrium of the ventricular apex at very low density and can be isolated by magnetic bead-based approaches [32, 33]. These cells are the first CSC population that have been successfully tested in clinical trials and demonstrated a profound increase in cardiac performance [17].

Cardiospheres are generated from the outgrowth of explants obtained from heart biopsies [34, 35]. Notably, due to their bigger size (up to 200 *μ*m), their administration via the intracoronary route (most common in CVDs) bears the risk of microembolization [36]. Yet, the improvement of cardiosphere-manufacturing methods was shown to reduce particle size to 50–100 *μ*m, making it suitable for a safe delivery via the coronary route in minipigs [37]. However, cardiosphere-derived SCs have been found to represent a better therapeutic product [38]. In the phase I CADUCEUS trial (NCT00893360), autologous cardiosphere-derived SCs, injected into patients suffering from ventricular dysfunction, led to reduced infarction size but lacked functional benefits [39]. Therefore, additional clinical trials need to be initiated to further validate the first promising results and to elucidate the entire regenerative potential of CSCs.

The cell selection in the case of ESCs and iPSCs mainly relates to the successful selection of pure cell populations after differentiation. This serves to ensure that tumorigenic, undifferentiated cells are not transplanted together with the final cell product. Several purification strategies have been established, such as surface marker isolation, manual enrichment, or density gradient centrifugation [40]. Cell enrichment based on the expression of a drug-resistant gene or fluorescent protein is a commonly used method that results in highly purified cell populations of ~95% [40, 41]. In contrast, flow cytometry in combination with antibodies, targeting specific surface markers for certain cell types (kinase insert domain receptor and platelet-derived growth factor receptor *α* for CSCs and signal-regulatory protein *α* for adult cardiomyocytes), represents another purification strategy which does not include genetic manipulation [42–45]. In addition, lactate-based enrichment and the application of nanosized probes to detect cell type-specific mRNA were used to generate iPSC-derived cardiomyocyte populations with a purity of more than 90% [46, 47]. In terms of clinical compatibility, it is so far preferred to apply purification approaches which preclude genetic modification.

In the only initiated clinical trial for CVD treatment using ESC-derived cardiac progenitors (purity of 99%), cells were transplanted into an infarct area of a patient with severe heart failure. As a result, at three months follow-up, no complications such as arrhythmias or tumor formation were observed, whereas an improvement of symptoms from New York Heart Association class III to I and an increase in LVEF of 10% were reported [9]. However, as promising as these results are, much better developed protocols, more data, and long-term proofs of safety are required to bring early progenitors to wide clinical practice [10, 14].

### 3.2. Improvement of SC Delivery

The outcome of cell therapy for cardiac regeneration strongly depends on the successful delivery of SCs to the site of interest. To date, two major routes of cell administration have been applied in preclinical and clinical studies: (1) systemic (intravenous) injection and (2) local (intramyocardial) transplantation [48]. Although intravenous injection is easy to apply and less invasive than local transplantation routes, injected SCs are widely distributed throughout the whole body and accumulate in the liver, lungs, and spleen [49, 50]. Moreover, this strategy mainly relies on the homing capacity and cell retention of the SC product [51]. Therefore, direct intramyocardial injection has been the most preferred method in preclinical and clinical trials [31]. Nevertheless, the engraftment of cells after delivery remains a very inefficient process. Studies in large and small animal models, for example, pigs, gave evidence that more than 90% of cells are washed out within the first hour after transplantation [48, 52–54]. Thus, the use of SCs for cardiac regeneration was accompanied by the development of appropriate equipment to ensure that the cell product reaches the target site. Several advanced delivery strategies were established to ensure minimally invasive and targeted cell delivery to the myocardium, including 3D MyoStar® Injection Catheter combined with NOGA® electromechanical mapping system, 2D fluoroscopic guidance systems Helix™ infusion catheter, and the MyoCath™ [55, 56].

#### 3.2.1. Application of Biomaterials

The additional application of biomaterials can enhance the delivery of cells into infarcted myocardium to a significant degree, accompanied by improved cell retention. For instance, the encapsulation of cells using hydrogels allows control over the microenvironment upon cell application. Embedding of CSCs into a hydrogel matrix profoundly increased long-term cell retention and cardiac regeneration three weeks after delivery into infarcted mice hearts [57]. Similarly, incorporation of cardiosphere-derived SCs into a hyaluronan-gelatin hydrogel led to a 3-fold enhancement of cell engraftment and an improvement of left ventricular ejection fraction (LVEF) and neovascularization [58]. Biomaterial-assisted cell delivery is performed either by injection of a SC matrix mixture into the heart or by transplantation of a cell-matrix patch. Both of these should be biocompatible and biodegradable and should form nontoxic degradation products [59]. The injection-based approach requires liquid biomaterials that solidify immediately after transplantation [59]. Patch-based strategies, in turn, imply the generation of tissue-like structures *in vitro* prior to transplantation [60, 61].

Importantly, these biomaterials can be additionally modified to contain functional molecules, beneficial for the therapeutic effect of delivered SCs, like insulin-like growth factor 1 (IGF-1), stromal-derived factor 1 (SDF-1), and transforming growth factor *β* (TGF-*β*) [48, 59, 62, 63]. Moreover, oxygen-releasing scaffolds have been developed to increase the O_2_ level at the site of transplantation for several hours to days, which further improves survival and proliferation of applied SCs [63–65]. Biomaterials like matrigel, cardiogel, fibrin, or collagen represent biodegradable scaffolds that support adhesion, differentiation, and proliferation of different types of SCs, including ESCs and bone marrow-derived SCs as demonstrated in small and large animals [48, 59]. For adipose-derived MSCs, it was shown that coinjection with fibrin increased cell retention by 50% four weeks after transplantation into murine hearts [66]. The supporting effect of biodegradable scaffolds has also been proven in a study with human MSCs that were incorporated into a collagen matrix and applied to rats with MI [67]. The authors showed that human MSC patch application led to improved diastolic properties and significantly enhanced the number of blood vessels in the peri-infarct area by 30%. Likewise, the application of cell-supporting synthetic scaffolds induces proangiogenic effects, as shown in murine hearts [68]. Chung and coworkers used vascular endothelial growth factor- (VEGF-) loaded poly-l-lactic acid as a vehicle to transplant CSCs into rat hearts [69]. The density of microvessels was significantly increased by ~25%, and a higher number of cardiomyocytes was determined within the infarcted heart tissue four weeks after cell injection. Another example is polyvinylidene fluoride-based scaffolds, which have been recently produced as vehicles for SC delivery in cardiac regenerative therapy. These scaffolds possess piezoelectric characteristics which may be beneficial especially for the application in cardiovascular tissue. *In vitro* studies with ESCs and ESC-derived cardiovascular cells already demonstrated the feasibility of polyvinylidene fluoride as a vehicle for SC delivery [70].

#### 3.2.2. Magnetic Cell Targeting

The concept of magnetically targeted delivery implies labeling or loading of SCs with particles responsive to a magnetic field in order to facilitate cell guidance to the area of interest. Using this approach, different research groups have demonstrated successful *in vitro* and *in vivo* results. For example, a study by Vandergriff and coworkers demonstrated that magnetic targeting can increase cell retention and engraftment of cardiosphere-derived SCs to the infarcted rat myocardium ~4-fold compared to control where no magnet was applied [71]. This enhancement of cell retention was accompanied by augmented angiogenesis, smaller scar size, and improved cardiac performance. Further *in vivo* studies showed an increased engraftment and functional benefits of magnetically labelled cardiosphere-derived SCs if compared to the nontargeted group [72, 73]. Similar improvement of cell retention was observed by Shen et al. 24 hrs after transplantation of MSCs in a rat MI model. However, a long-term analysis of these animals (three weeks) showed less pronounced differences between magnetically targeted and nontargeted cells [74]. Interestingly, the data also suggested that too high magnetic intensity can cause microembolization and hamper the positive effect on cardiac performance [74]. Another promising study has been published by Cheng and coworkers in 2014 [75]. Superparamagnetic iron nanoparticles were simultaneously conjugated with two antibodies targeting CD45^+^ therapeutic endogenous SCs and injured cardiomyocytes [75]. After intravenous injection of these particles into the injured myocardium of rats and local application of a magnetic field, targeting of CD45^+^ cells to the infarcted region was enhanced 10-fold, as well as their therapeutic activity [75]. Importantly, as various studies in small and large animals have demonstrated, magnetic nanoparticles are also applicable to track cells via MR imaging [76–79].

To date, magnetic particles have been used in several *in vitro* and preclinical studies [80–84]. However, some safety concerns need to be addressed before wide clinical translation. In particular, increased iron concentrations can increase the intracellular level of free radicals (in a dose-dependent manner) [84]. Moreover, the application of strong magnetic fields can have enhancing or inhibiting effects on biological systems or lead to the formation of toxic aggregates from intracellularly located magnetic particles [84–86]. In addition, certain limitations should be taken into account when using magnetic nanoparticles for imaging of transplanted SCs: it is difficult to distinguish between magnetized viable and dead cells; and long-term MRI-based follow-up of injected cells is compromised due to the leakage of iron particles or their uptake by macrophages [23, 77, 87]. At the same time, the use of MR reporter genes might help to overcome this problem. Overexpression of the transferrin receptor or ferritin are commonly used to augment the intracellular iron concentration and profoundly enhance contrast in MRI tracking [88, 89].

#### 3.2.3. Ultrasound-Mediated Delivery of SCs

The application of microbubbles to tag cells followed by ultrasound-mediated cell targeting is a novel technique that can significantly promote cell retention and engraftment at the site of injury. In this concept, gas-filled microbubbles are attached to the SCs, which thereby become highly susceptible to acoustic radiation forces. Thus, SCs can be placed and arrested at the injured area using ultrasound catheter intracoronary injection [90, 91]. In particular, in a rabbit model, the application of microbubble-tagged MSCs and ultrasound led to a 150-fold enrichment of cells at the endoluminal surface [90]. Such enhanced efficiency of cell delivery via microbubble/ultrasound system was confirmed in a large animal model [92]. In this study, the increased MSC engraftment was accompanied by a slight but significant improvement of cardiac functions and cardiac remodeling after MI in dogs [92]. Moreover, Woudstra and colleagues designed microbubbles coated with antibodies, targeting both the SC-specific marker CD90 and an adhesion molecule expressed on endothelial cells within the infarcted area. This experimental setup allowed specific delivery to the damaged myocardium in a rat MI model, while almost no cells were found in the noninfarcted area [93].

### 3.3. Nongenetic Modification to Improve SC Efficiency

Once SCs are delivered to the infarcted area, long-term survival and engraftment are prerequisites for sufficiently exerting their therapeutic activity and to establish successful clinical treatments. Numerous ex vivo manipulation strategies have been employed to increase survival, homing, and engraftment of injected SCs [48, 63, 94]. Nongenetic approaches and genetic cell engineering are applied to generate a “second generation” of SC therapeutics, which should come close to the ultimate goal of regenerative medicine to renew defected cardiac tissue with new functional cells.

#### 3.3.1. Hypoxic Pretreatment of SCs

The harsh microenvironment within damaged host cardiac tissue is one of the major obstacles for transplanted SCs. Low oxygen levels, deprived nutrient supply, oxidative stress, and inflammatory mediators impede successful engraftment and lead to cell death early after transplantation [95]. Hypoxic priming of SC prior to transplantation was found to stimulate endogenous cell defense mechanisms, thereby increasing cell survival and improving the beneficial effects of SC therapy [96–98]. In numerous preclinical studies, duration of hypoxia varied from hours to days, while the level of hypoxia commonly ranged between 0.5% and 3% [96, 99]. In the study of Hosoyama et al., transplantation of hypoxia-preconditioned cardiosphere-derived SC sheets into infarcted mice hearts improved left ventricular function and decreased infarction size, compared to SCs that were cultured under normoxic conditions [99]. Similar results were previously observed by other researchers that have applied hypoxia-preconditioned SCs to murine MI-treated hearts [96, 100]. In a large animal model, application of MSCs subjected to hypoxia for one day resulted in a significant increase of ventricular function and capillary density in infarcted pig hearts [101]. Apart from the positive impact on cardiac function and tissue regeneration, hypoxia was also found to improve cell engraftment, leading to 30% increase in the amount of SCs retained in the ischemic area [101].

The underlying mechanisms mediating the positive effects of hypoxic preconditioning are diverse. In terms of cell engraftment, it was shown that hypoxia increases the expression of CXC chemokine receptor 4 (CXCR4), a receptor involved in cell homing [102–104]. As a result, SC migration to the infarcted tissue was profoundly augmented *in vivo*, which was also demonstrated in numerous *in vitro* studies [103, 105–108]. Moreover, the upregulation of prosurvival and antiapoptotic factors facilitates cell survival after injection [96, 109, 110]. This higher cell viability is also supported by a lower level of damaging reactive oxygen species that was observed when cells were subjected to decreased oxygen levels [100]. In addition, hypoxia activates many signaling pathways, such as AKT or MAPK, leading to increased secretion of paracrine factors that contribute to cardiac regeneration [111–113].

#### 3.3.2. Preconditioning with Pharmacological Agents

Pretreatment of cells with pharmacological agents is another simple and cost-effective approach to improve their therapeutic activity. Drug-mediated preconditioning promotes the release of certain paracrine factors, including SDF-1, hepatocyte growth factor (HGF), or IGF which, in turn, are advantageous for the regeneration after cardiac injury [110, 114, 115]. Furthermore, certain chemical molecules can possess antiapoptotic properties increasing therefore the survival of applied SCs. In particular, pharmacological activation of Rap1, a GTP-binding protein, was found to improve survival and adhesion of transplanted MSCs and restore function of MI-treated rat hearts [116]. Similarly, selective activation of the cannabinoid receptor type two in injected adipose-derived SCs positively influenced the remodeling process and improved cardiac functions in mice, probably by enhancing paracrine signaling of SCs and resistance to oxidative damage [117].

Paracrine signaling was proven to be one of the major mechanisms mediating the regenerative capacity of SCs. At the same time, the direct conversion into cardiomyocytes also contributes to the benefits provided by SC therapy [118–120], and pharmacological treatment was shown to facilitate the myogenic differentiation. For example, the DNA demethylating agent 5-azacytidine was extensively described to enhance the differentiation of SCs into cardiac-like cells *in vitro* [121–124]. Moreover, preincubation with 5-azacytidine significantly promoted the cardiogenic differentiation capacity of MSCs when transplanted into pig hearts, although a positive effect on cardiac performance was not detected [125]. The latter is in line with a report by Mykhaylichenko et al., where 5-azacytidine-modified SCs did not profoundly improve cardiac function and morphological parameters, for example, size of infarction area [126]. Thus, novel pharmacological strategies need to be established to promote the capacity of SCs for cardiac lineage specification. However, even if applied SCs demonstrate cardiac-specific markers after transplantation, successful integration into the host myocardium is required in order to significantly enhance contractility.

Notably, application of chemical compounds also plays an emerging role in SC-based generation of cardiac cells by stimulation or inhibition of cellular signaling pathways such as Wnt or bone morphogenetic protein (BMP) [127–129]. These small molecule-mediated programming/reprogramming strategies help to improve the quality of *in vitro*-produced cardiac cells suitable for the transplantation into ischemic hearts to replace damaged tissue [130, 131].

#### 3.3.3. Application of Growth Factors and Cytokines

In addition to pharmacological agents, growth factors and cytokines are powerful molecules to influence SC activity. Several molecules have been shown to determine cell fate towards the cardiogenic lineage. For instance, fibroblast growth factors (FGF) or BMP4 are promising compounds that promote the differentiation of SCs into cardiac-like cells or cardiomyocytes and thus can be applied to prime cells before injection [132]. In particular, in a phase II C-CURE clinical trial (NCT00810238), lineage-guided MSCs were found to be safe and beneficial in chronic heart failure [133]. In this case, MSCs were exposed ex vivo to various growth factors and cytokines (TGF-*β*, BMP4, FGF, etc.) mimicking natural cardiogenic conversion prior to transplantation. As a result, a significantly improved LVEF and six-minute walk distance were demonstrated, which highlight the potential of lineage-guided SCs for the treatment of ischemic heart failure [133].

Since growth factors and cytokines are key players in cellular physiology, they have been used to manipulate different signaling pathways in order to modify SC properties apart from cell fate commitment. For example, incubation with SDF-1 before injection enhanced the capacity of endothelial progenitor cells to promote angiogenesis, indicated by increased network formation *in vitro* [134]. As shown by Pasha et al., the transplantation of SDF-1 primed bone marrow-derived MSCs in rats suffering from MI significantly improved cardiac performance and cardiac remodeling as indicated by reduced infarction size and fibrosis [135]. Additional administration of a CXCR4 agonist abolished the observed positive effect of MSCs on myocardial repair. Moreover, an enhancement of SC efficiency in rats was also observed following TGF-*α* treatment, leading to a greater postischemic myocardial functional recovery compared to untreated cells [136]. The authors suggested that the improved efficiency of applied SCs is based on a reduced myocardial production of proinflammatory cytokines and on the TGF-mediated upregulation of VEGF in preconditioned MSCs [136].

### 3.4. Genetic Modification to Improve SC Efficiency

In contrast to nongenetic approaches, genetic modification is another concept to boost the potency of SC products. In general, four main strategies of genetic modification can be applied: protein overexpression by DNA delivery, gene silencing (e.g., by RNAi), gene editing (TALENs, CRISPR/Cas9), and miRNA-based modifications [137, 138].

#### 3.4.1. DNA-Based Cell Modification

Since the paracrine activity of SCs is of great importance for their regenerative capacity [2], the overexpression of therapeutic factors can be induced, which are normally released by the cell to support cardiac regeneration upon ischemia (VEGF, HGF, IGF, SDF-1, FGF, etc.) [95, 139]. For instance, viral transduction of adipose-derived MSCs with an IGF-1 construct enhanced the release of IGF, VEGF, and HGF and improved the ejection fraction 6 weeks after cell injection into rats with MI [140]. However, despite the fact that IGF-1 was shown to have an antiapoptotic effect on cells transplanted in the ischemic environment, no transplanted cells were detected at this time point [141]. This indicates that long-term cell survival in this experimental setup was not improved by IGF-1 overexpression. In a similar study, Gómez-Mauricio and coworkers induced overexpression of both IGF-1 and HGF in adipose-derived pig SCs followed by their injection in pig MI model [142]. Animals treated with these modified cells showed reduced inflammation and improved angiogenesis, although no beneficial effect on cardiac function parameters were detected.

To increase SC attachment to the extracellular matrix of the host tissue, Li et al. selected integrin *β*1 as a target protein for overexpression studies [143]. As a result, echocardiography of MI-treated mouse hearts indicated a ~25% improvement of cardiac performance as well as SC survival one week after transplantation. This is in accordance with previous observations of Mao et al. showing that manipulating integrin signaling pathway is a suitable tool for promoting the therapeutic outcome of MSC transplantation in pigs, including lower degree of fibrosis, increased myocardial perfusion, and microvessel density [144]. Other promising targets, which also have demonstrated a positive effect on SC viability, are the apoptosis-regulating protein BCL-2 and the channel-forming connexin43 [95, 145]. Moreover, overexpression of several proteins, including NKX2.5, TNNIK, hypoxia-inducible factor-1, CXCR4, and AKT1, has been proven to enhance homing, survival, and differentiation of SC [95, 146, 147].

Taken together, the data shows that the overexpression of proteins in SCs can significantly improve their efficiency and support cardiac regeneration. At the same time, the most commonly applied method for DNA delivery to cells is viral transduction—mainly due to its high efficiency [148, 149]. Yet, in terms of clinical translation, the use of viruses is suboptimal due to safety issues, including mutagenesis, tumorigenesis, and potential immune reaction of the host. This could be solved via nonintegrating (incl. nonviral) delivery systems. Likewise, the use of DNA itself as a therapeutic molecule carries similar risks as it can be randomly inserted, possibly leading to malignant transformation. One strategy to reduce undesired activation of oncogenic genes is the application of the novel CRISPR/Cas9 gene editing technology that allows precise insertion of therapeutic genes into the SC genome without causing a dysfunction of neighboring genes [137, 138].

#### 3.4.2. miRNA-Based Cell Modification

miRNAs are small 20–25-nucleotide-long noncoding RNAs that regulate gene expression on mRNA level. Since their discovery in 1993, miRNAs have been identified to play a crucial role in various cellular processes, including development, cell fate commitment, proliferation, and cell signaling [150–153]. In cardiac regeneration, the ability of miRNAs to promote SC survival by increasing the resistance to high oxidative stress was demonstrated for let-7b [154]. By targeting caspase-3, let-7b regulated apoptosis and autophagy in MSCs. Three days after injection, the number of cells that resided in the infarcted heart was twice higher compared to unmodified cells. Moreover, cardiac function was restored [154]. Likewise, miR-133, miR-126, and miR-301 engineered SCs exhibit an improved survival and engraftment when transplanted into MI-treated hearts [94, 155, 156].

In addition to prosurvival and homing activity, ex vivo modification of SCs with miRNA was also applied to modulate their paracrine activity. For example, following transfection with miR-146 of MSCs, the expression and paracrine release of VEGF were enhanced by 300%. These, in turn, promoted the angiogenic effect *in vitro* and *in vivo*, leading to reduced fibrosis and improved ejection fraction in murine hearts [157]. Likewise, miR-126 and miR-377 were identified as promising candidates to modulate the release of VEGF in cells prior to transplantation [158, 159]. In contrast to the use of described miRNA mimics, enhancement of the angiogenic capacity of hiPSCs was also achieved when miR-495 was inhibited. As a result, an increased neovascularization in the infarcted heart was observed as well as integration of SCs to coronary vessels [160].

As mentioned earlier, transdifferentiation of transplanted cells contributes to the regenerative capacity of SCs [118–120]. In this case, the modulation of miRNA expression is beneficial to trigger a cardiac cell fate. This was investigated in a large number of *in vitro* studies. In particular, combination of 5-aza treatment and miR-1-2 overexpression provoked the activation of cardiac-specific genes in MSCs via the Wnt-signalling pathway [161, 162]. Similarly, cardiac lineage specification of ESCs was enhanced upon miR-1 overexpression [163]. In addition, the let-7 family was found to stimulate the maturation of ESC-derived cardiomyocytes [164].

Compared to DNA-based approaches, miRNA application offers the possibility to induce transient effects which improve the therapeutic properties of SCs. Since no alterations of the genome are required, this epigenetic modification is likely favorable for future clinical translation. However, as miRNAs could have multiple targets, off-target effects need to be addressed when applied as SC modifiers. In addition, expression of the same gene can be regulated by several miRNAs and their possible compensation should be accounted.

A selection of improvement strategies applied in clinical trials and *in vivo* studies is illustrated in Table 1.

### 3.5. Tissue Preconditioning

Injured tissue preconditioning is a complementary method to the preconditioning strategy of transplanted stem cells. The main reason of this approach is to produce a more favourable microenvironment for the applied stem cells, leading to improved cell engraftment [176]. It was reported that ischemic postconditioning can increase the beneficial effects of MSC transplantation by improving engraftment and cell survival [177, 178]. More recently, it was discovered that these positive effects of MSC injection were mainly attributed to the hospitable environment [179]. Likewise, pharmacological pretreatment with statins was found to promote the survival and the therapeutic effects of bone marrow and adipose-derived SCs on damaged myocardium [180, 181]. Additionally, physical cues were utilized to make the transplant site more susceptible to donor cells. The application of low-energy shock waves was shown to increase the expression of chemoattractant factors in a rat model of chronic limb ischemia, resulting in an enhanced recruitment of transplanted endothelial progenitors [182]. As a recent report indicated on the clinical trial CELLWAVE, pretreatment with shock waves increased cell homing of injected bone marrow-derived mononuclear cells to the target area and promoted the outcome of cell therapy [170].

## 4. Strategies to Improve MSCs

Among the cell types used as cell therapeutics in cardiac regeneration, MSCs are one of the most attractive for several reasons. First, MSCs can be easily isolated from different tissues, including bone marrow, peripheral blood, umbilical cord, and adipose tissue [148]. Next, they can be amplified *in vitro* and subjected to genetic and nongenetic cell engineering modifications, although possible disadvantages of ex vivo culturing should be accounted (see Section 2.1) [95]. The regenerative potential of MSCs has been proven by now, whereas the mechanistic basis of it is still under investigation. To date, their capacity for multilineage differentiation has been demonstrated, as well as the ability to control SC niches (in HCS and bone marrow) and the secretion of proangiogenic paracrine factors (VEGF, basic FGF, and PDGF) [183]. In addition, an immunomodulatory and immunosuppressive activity of MSCs has been well described both *in vitro* and *in vivo* [148, 184]. At the same time, the reports on MSC transdifferentiation to cardiomyocytes are controversial.

Due to all beneficial properties characterizing MSCs, a lot of progress has been made to bring them from bench to bedside. Even though safety and feasibility of allogenic and autologous MSC transplantation has been shown in several clinical trials, the conclusions regarding their efficiency and therapeutic outcome differ [15, 148, 185] (Table 2). Therefore, it is of particular importance to develop clinically relevant improvement strategies which can be utilized to modify MSCs.

Among the previously mentioned engineering approaches applied to MSCs (Sections 3.1–3.4), genetic modification is likely the most promising one, mainly because of its multimodality and ability to cover several challenges for SC therapy, simultaneously [186]. For example, the introduction of reporter genes allows isolation of highly purified cell populations by flow cytometry or the tracking of transplanted cells (e.g., as a result of expression of fluorescent or luminescent proteins) [187, 188]. Moreover, forced expression of certain factors with high cardiogenic potential can be achieved in transplanted cells [189]. In addition, introduction of factors, which are responsible for such innate cell properties as mobilization, adhesion, migration, or integration, can enhance MSC retention and activity in the desired area [143, 190, 191]. A similar outcome can be achieved by enhancing intrinsic cell properties, that is, their survival in an ischemic environment or their paracrine potential [145, 191, 192]. Furthermore, a broad spectrum of therapeutic agents can be incorporated in order to specifically complement and promote regenerative properties of delivered cells [159, 193].

### 4.1. Improvement of MSC Resistance: Induction of Prosurvival Proteins

A large amount of *in vitro* data and preclinical studies indicated that MSCs overexpressing therapeutic molecules showed higher potency in the treatment of CVDs. For example, Akt-modified bone marrow-derived MSCs exhibited an increased survival in the myocardium of murine hearts up to two weeks after transplantation [95, 110]. Similar beneficial effects on MSCs survival *in vivo* and *in vitro* were induced by overexpression of antiapoptotic genes such as BCL-2 or heat shock proteins [110, 194]. Likewise, IGF-1 transformed MSCs exhibited an increased intracellular level of prosurvival factors, inhibiting cell death after transplantation into ischemic hearts [95, 149]. A lentiviral-mediated overexpression of integrin *β*1 profoundly decreased proapoptotic proteins in MSCs, including caspase 3 and Bax, which, in turn, led to improved cell survival one week after intramyocardial injection into rat heart [143].

Programmed cell death of transplanted MSCs is also triggered by the hypoxic conditions prevailing in the infarcted heart tissue. In particular, HGF-1 was found to increase the resistance of overexpressing MSCs to low oxygen levels and restore heart function in a mouse model [195]. Moreover, miRNA-based reprogramming could improve cell survival. Dakhlallah and coworkers engineered MSCs by introducing miR-133a, which decreased the expression of proapoptotic genes and resulted in a 2-fold improvement of cell engraftment one week after injection in MI-treated rat hearts [155]. In addition, overexpression of miR-1, miR-23a, and miR-210 impede cell death and prolong survival *in vivo* and *in vitro* [95].

### 4.2. Improvement of Adhesion and Engraftment: Induction of Homing Factors and Cell-Matrix Interaction

Once delivered to the damaged heart tissue, the homing and engraftment of MSC is rather low. Since one of the major regulators of SC homing *in vivo* is the SDF-1/CXCR4 signaling axis, MSCs overexpressing CXCR4 demonstrated a 2-fold enhancement of their homing capacity when compared to untreated MSCs [196]. In line with this, a higher level of SDF-1 and CXCR4 was observed in protein kinase C overexpressing MSCs, which, in turn, resulted in an increased number of retained cells in infarcted rat hearts that was twice higher compared to control MSCs [190]. Similarly, the stimulation of CXCR4 expression and MSC homing was also documented for interleukin 6 [197].

On the other hand, cell-cell contacts and cell-matrix interactions are important for proper adhesion and engraftment. One group of key molecules mediating cell-matrix adhesion and participating in signal transduction are proteins of the integrin family [198]. Thus, targeting integrin-linked kinase was found to markedly augment homing and regenerative capacity of intracoronary-injected MSCs in minipigs [199]. After two weeks, the authors detected a 4-fold higher number of MSCs overexpressing integrin-linked kinase [199]. Moreover, a novel promising cytokine that promotes cell engraftment of MSCs was recently identified by Bortolotti and colleagues. They used an *in vivo* functional screening approach and found that cardiotrophin 1 increased persistence of injected MSCs and preserved cardiac function [200].

Apart from protein overexpression, engraftment of transplanted cells and their homing to injured tissue can be regulated by miRNA-based modifications: for the first purpose, miR-133a, miR-126, miR-34a, and combination of miR-21, miR-24, and miR-221 were reported to be efficient, miR-150, miR-146, and miR-15a/16—for the latter [155, 201].

### 4.3. Improvement of Vascularization and Cardiac Remodeling: Induction of Proangiogenic Factors and miRNA

The formation of new blood vessels within the infarcted area is of particular importance for restoring cardiac performance. MSCs can support angiogenesis by the following mechanisms: (1) release of paracrine factors stimulating vessel formation, (2) differentiation into endothelial or vascular smooth muscle cell linage, and (3) acting as perivascular cells [148]. All of these functions can be promoted by suitable cell modification.

VEGF is one of the key factors regulating neovascularization and *in vivo* studies using VEGF overexpressing MSCs showed improved angiogenic potential by 30% in rat and mice models [202–204]. Another signaling cascade shown to be crucial in the mediation of the proangiogenic influence of MSCs is phosphoinositide 3-kinase-Akt signaling. Therefore, targeting this pathway by overexpression of VEGF, HGF, or IGF led to improved vascularization, contractility, and reduced infarction size and cardiac remodeling in murine MI models [95, 205].

miRNA-based modification can also be applied to promote the proangiogenic properties of MSCs. Recently, it was shown that transfection of MSCs with miR-146a augments the secretion of VEGF. Compared to the untreated MSCs, animals treated with miR-146a-MSCS exhibited a 50% lower degree of fibrosis and a significantly enhanced ejection fraction [157]. Likewise, a positive effect on angiogenesis and heart function was shown for the proangiogenic miR-21 and miR-126 [206–208].

In addition, in terms of influencing cardiac remodeling, MSCs engineered to express heme oxygenase-1 or thioredoxin-1, an antioxidant and regulator of transcription factors and cytokines, displayed increased cardioprotective effects [209, 210].

Notably, the concept of cell modification to augment the therapeutic value of MSCs is strongly supported by the C-CURE phase II clinical trial, where priming of hMSCs by a cytokine cocktail was performed in order to obtain cardiopoietic lineage-specified cells [133]. This has proven the safety of cell modification and its feasibility, which also resulted in the initiation of a similar trial CHART-1 (NCT01768702) [171]. At the same time, the impact of genetically modified MSCs on cardiac regeneration has not yet been studied in patients. However, first clinical phase I/II studies of such sort have been established for the treatment of gastrointestinal tumors and lung cancer [211, 212]. In these cases, to induce antitumor effects, MSCs are modified by viral vectors to produce anticancer therapeutics that are released by the cell after homing to the tumor site. For cardiac patients, the safety and benefits of genetic engineering of MSCs still have to be balanced and extensively studied.

### 4.4. Improvement of MSC-Derived Exosomes: Reduction of Fibrosis and Inflammation

The beneficial paracrine effects of transplanted MSCs are, in part, mediated by the release of exosomes. These are extracellular vesicles 30–100 nm in diameter, which contain a variety of molecules, including proteins, miRNA, and mRNA, and thereby play an important role in cell-cell communication [213, 214]. Several preclinical studies have demonstrated the benefits of exosome administration in the treatment of CVDs. A reduction of the infarction area by 50% was achieved when exosomes isolated from MSCs were injected into infarcted rat hearts. In addition, this exosome-based treatment promoted neoangiogenesis by up to 40% and decreased the infiltration of inflammatory cells into the infarcted ventricular tissue [215]. *In vivo* data obtained by other groups confirmed that MSC-derived exosomes support vessel formation, inhibit the cardiac remodeling process, and preserve pump function of the injured heart [216–219]. Interestingly, in a comparative study, effectiveness of exosomes was found to be superior to MSC injection in a rat model of MI, showing that cardiac fibrosis and inflammation, as well as cardiac performance, were significantly improved in exosome treated hearts [220]. In addition, a meta-analysis by Zhang and colleagues confirmed the benefits of exosome administration on cardiac regeneration [221].

Importantly, cell engineering-based modifications of MSCs have also been demonstrated to favorably influence the cardioprotective properties of released exosomes [222]. This could be used as a tool to further promote the positive outcome of exosome injection. For example, exosomes isolated from Akt overexpressing MSCs induced a 2-fold enhancement of neovascularization in rat hearts which was reflected in a profound improvement of LVEF [223]. Accordingly, *in vitro* data showed that these exosomes significantly augment the proliferation, migration, and network formation capacity of endothelial cells [223]. In a previously published study, a similar promoting effect on angiogenesis *in vivo* was described for exosomes derived from MSCs overexpressing hypoxia inducible factor-1*α* [224]. Likewise, purified exosomes released from MSCs overexpressing CXCR4, and GATA4, were found to have a higher potential for cardioprotection compared to exosomes derived from normal MSCs [225, 226]. Exosome-mediated cell-free therapy for the treatment of CVDs has not been applied in clinical trials yet. However, phase I/II studies for cancer therapy using exosomes already confirmed its general safety [219, 227].

## 5. Conclusion

The strategy of using SCs for the treatment of CVDs was considered to be the most promising approach for heart regeneration, intended to complement or replace currently existing clinic treatment options. Indeed, numerous preclinical studies have demonstrated the strong regenerative potential of SCs. However, due to the inconsistent results from clinical trials and the low efficiency of transplanted SCs, this concept could not fully meet the expectations and be widely integrated into clinical practice. Since the therapeutic potential of SCs is undisputable, researchers have made considerable effort to significantly improve the effectiveness of SCs by the generation of modified cell products.

Although the efficiency of modified SCs has been shown to be superior to unmodified cells *in vitro* and *in vivo*, most studies focused on one single strategy to improve the therapeutic outcome. However, cardiovascular disorders are complex diseases with multiple mechanisms involved in pathogenesis. Therefore, curative concepts with complex activity are required. Thus, while improving cell-based therapeutics, it is crucial to take into account that multiple features should be facilitated simultaneously. For example, it is not sufficient to improve cell survival or retention, but also, a profound proangiogenic and cardioprotective activity should be ensured, and age-related decline of SC efficiency should be eliminated. Several strategies could be used to follow this principle, including genetic cell modification, cytokine preconditioning, and pharmacological treatments or their hybrids.

In order to achieve the highest possible outcome in SC treatment, a system of patient's response predictors should be developed. It has been previously shown that patients with worse baseline condition responded to bone marrow-derived mononuclear cell therapy, whereas others with better health condition did not [228, 229]. Therefore, a system is required that allows classification and selection of patients matching cell therapeutics. Plasma profiling of patients could help to find novel biomarkers that identify responders and nonresponders [230]. Recently, in the phase III PERFECT clinical trial, the nonresponse on endothelial progenitor cell administration was found to be associated with the expression of SH2B3 protein [165].

To summarize, the whole concept of SC modification has already been proven to be feasible and safe in clinical trials using MSCs [231, 232]. Extensive work is still needed to generate powerful off-the-shelf SC therapeutics. Together with personalized cell-based therapy (e.g., responders versus nonresponders), SCs might fulfill the expectations of novel curative options for cardiac patients.

## Figures and Tables

**Table 1:** Selection of clinical trials applying stem cell therapeutics for CVD treatment and examples of developed improvement strategies.

Cell type	Clinical trials in CVD treatment	Main effect of cell therapy	Improvement strategy (indicated whether tested in clinical trials or *in vivo*)	Main outcome of improvement strategy
HSCs and EPCs	PERFECT [165]	No improvement (slight improvement in responder group)	*In vivo*: delivery by polymer micro-bundle scaffold [166]	Enhanced cell survival and retention
	REGENT [167]	Slight improvement	*In vivo*: pretreatment with deacetylase inhibitor [168]	Enhanced CXCR4 and VEGF level, increased vessel formation in ischemic muscle
	ACT34-CMI [169]	Improved exercise tolerance		

MSCs			Clinical trial (CELLWAVE): cardiac shock wave pretreatment [170]	Improved retention, increased LVEF and cardiac remodeling
	C-Cure [133]	Improvement of LVEF and 6 min walk distance	Clinical trial: (C-CURE; CHART I/II) cell preconditioning with procardiogenic cytokines	Increased LVEF
	Chart I/II [171]	No improvement, ongoing (CHART II)	*In vivo*: ultrasound-mediated delivery [92]	Increased engraftment, improved cardiac remodeling and function
			*In vivo*: pharmacological activation of Rap1 [116]	Improved homing capacity and cardiogenic differentiation, increased cardiac performance
			*In vivo*: miR-146-based modification [157]	Augmented VEGF secretion, improved cardiac remodeling and angiogenesis, improved heart function

CSCs	SCIPIO [17]	Improvement of LVEF, reduced infarct size	*In vivo*: injection with VEGF-loaded scaffold [69]	Enhanced microvessel formation
	ALLSTAR (NCT01458405)	Ongoing	*In vivo*: magnetic-based delivery [71]	Increased cell retention and angiogenesis
	CAREMI [172]	Ongoing	*In vivo*: hypoxic preconditioning [99]	Increased LVEF, decreased infarction size

CDCs	PERSEUS [173]	Reduced scar size, improved LVEF	*In vivo*: encapsulation into hydrogel [57]	Increased cell retention and LVEF, augmented angiogenesis
	CADUCEUS [174]	Reduced scar size	*In vivo*: magnetic targeting [72]	Enhanced cell retention and engraftment, reduced scar size

ESCs	1 patient (application of ESC-derived cardiac progenitors) [9]	Improvement of LVEF and 6 min walk distance	*In vivo*: cells loaded on fibrin scaffold [175]	Improved cardiac function, enhanced angiogenesis
	ESCORT (NCT02057900)	Ongoing (recruiting)		

iPSCs	Not yet tested in clinical trials for CVDs		*In vivo*: modification with miR-495 mimic [160]	Enhanced angiogenesis

CVDs: cardiovascular diseases; HSCs: hematopoietic stem cells; ESCs: endothelial stem cells; CXCR4: C-X-C chemokine receptor 4; VEGF: vascular endothelial growth factor; MSCs: mesenchymal stem cells; LVEF: left ventricular ejection fraction; Rap1: Ras-proximate-1; CSCs: cardiac stem cells; CDCs: cardiosphere-derived SC; ESCs: embryonic stem cells; iPSCs: induced pluripotent stem cells.

**Table 2:** Examples of clinical studies applying MSCs for the treatment of chronic and acute CVDs.

Clinical study	MSC type	Cardiac disease	Modification	Applied cell number	Route of administration	Time of MSC application after MI	Effect on LVEF
POSEIDON [233]	Bone marrow MSCs (allogenic versus autologous)	Nonischemic dilated cardiomyopathy		1 × 10^8^	Transendocardial		No change
RIMECARD [234]	Umbilical cord MSCs versus bone marrow MSCs (allogenic)	Dilated cardiomyopathy		1 × 10^6^/kg	Intravenously		Improved
PROMETHEUS [15]	Bone marrow MSCs (autologous)	Chronic ischemic cardiomyopathy		2 × 10^7^–2 × 10^8^	Intramyocardial		Improved
MSC-HF [235]	Bone marrow MSCs (autologous)	Ischemic heart failure		~8 × 10^7^	Intramyocardial		Improved
C-CURE [133]	Bone marrow MSCs (autologous)	Chronic heart failure	Preconditioning with cardiogenic cytokines	7 × 10^8^	Endoventricular		Improved
Xiao et al. [236]	Bone marrow MSCs versus bone marrow MNCs (autologous)	Dilated cardiomyopathy		~5 × 10^8^	Intracoronary		Improved
Perin et al. [237]	Bone marrow MSCs (allogenic)	Chronic heart failure		2.5–15 × 10^7^	Transendocardial		No change
HUC-HEART trial [238]	Umbilical cord MSCs (allogenic) versus bone marrow BMNCs (autologous)	Chronic ischemic cardiomyopathy		2–20 × 10^7^	Intramyocardial		Improved
Buttler et al. [239]	Bone marrow MSCs (allogenic)	Nonischemic cardiomyopathy	Hypoxic preconditioning	1.5 × 10^6^/kg	Intravenously		No change
TAC-HFT [240]	Bone marrow MSCs versus bone marrow MNCs (autologous)	Chronic ischemic cardiomyopathy		2 × 10^8^	Transendocardial		No change
MESAMI 1 [241]	Bone marrow MSCs (autologous)	Ischemic cardiomyopathy		4–10 × 10^7^	Intramyocardial		Improved
Anastasiadis et al. [242]	Bone marrow mesenchymal precursor (allogenic)	Chronic ischemic cardiomyopathy		1–4 × 10^6^	Intramyocardial		Improved
Chen et al. [243]	Bone marrow MSCs (autologous)	Acute myocardial infarction		48–60 × 10^9^	Intracoronary	~3 weeks	Improved
Lee et al. [244]	Bone marrow MSCs (autologous)	Acute myocardial infarction		~7 × 10^7^	Intracoronary	~4 weeks	Improved
Gao et al. [245]	Bone marrow MSCs (autologous)	Acute myocardial infarction		~3 × 10^6^	Intracoronary	~2 weeks	No change
Chullikana et al. [246]	Bone marrow MSCs (allogenic)	Acute myocardial infarction		2 × 10^6^/kg	Intravenously	~2 days	No change
Wang et al. [247]	Bone marrow MSCs (autologous)	Acute myocardial infarction		2 × 10^8^	Intracoronary	~3 weeks	No change
Rodrigo et al. [248]	Bone marrow MSCs (autologous)	Acute myocardial infarction		3–50 × 10^6^	Intramyocardial	~3 weeks	Improved
Gao et al. [249]	Umbilical cord MSCs (allogenic)	Acute myocardial infarction		6 × 10^6^	Intracoronary	~1 week	Improved

MSCs: mesenchymal stem cells; LVEF: left ventricular ejection fraction; MI: myocardial infarct.
